# Factor XIII Transglutaminase Supports the Resolution of Mucosal Damage in Experimental Colitis

**DOI:** 10.1371/journal.pone.0128113

**Published:** 2015-06-22

**Authors:** Christina Andersson, Peter H. Kvist, Kathryn McElhinney, Richard Baylis, Luise K. Gram, Hermann Pelzer, Brian Lauritzen, Thomas L. Holm, Simon Hogan, David Wu, Brian Turpin, Whitney Miller, Joseph S. Palumbo

**Affiliations:** 1 Novo Nordisk A/S, Biopharmaceutical Research Unit, Copenhagen, Denmark; 2 Division of Allergy and Immunology, Cincinnati Children’s Hospital Medical Center, Cincinnati, OH, United States of America; 3 Cancer and Blood Diseases Institute, Cincinnati Children’s Hospital Medical Center, Cincinnati, OH, United States of America; Duke University Medical Center, UNITED STATES

## Abstract

The thrombin-activated transglutaminase factor XIII (FXIII) that covalently crosslinks and stablizes provisional fibrin matrices is also thought to support endothelial and epithelial barrier function and to control inflammatory processes. Here, gene-targeted mice lacking the FXIII catalytic A subunit were employed to directly test the hypothesis that FXIII limits colonic pathologies associated with experimental colitis. Wildtype (WT) and FXIII^-/-^ mice were found to be comparable in their initial development of mucosal damage following exposure to dextran sulfate sodium (DSS) challenge. However, unlike FXIII-sufficient mice, FXIII-deficient cohorts failed to efficiently resolve colonic inflammatory pathologies and mucosal damage following withdrawal of DSS. Consistent with prior evidence of ongoing coagulation factor activation and consumption in individuals with active colitis, plasma FXIII levels were markedly decreased in colitis-challenged WT mice. Treatment of colitis-challenged mice with recombinant human FXIII-A zymogen significantly mitigated weight loss, intestinal bleeding, and diarrhea, regardless of whether cohorts were FXIII-sufficient or were genetically devoid of FXIII. Similarly, both qualitative and quantitative microscopic analyses of colonic tissues revealed that exogenous FXIII improved the resolution of multiple colitis disease parameters in both FXIII^-/-^ and WT mice. The most striking differences were seen in the resolution of mucosal ulceration, the most severe histopathological manifestation of DSS-induced colitis. These findings directly demonstrate that FXIII is a significant determinant of mucosal healing and clinical outcome following inflammatory colitis induced mucosal injury and provide a proof-of-principle that clinical interventions supporting FXIII activity may be a means to limit colitis pathology and improve resolution of mucosal damage.

## Introduction

The plasma transglutaminase factor XIII (FXIII) is most well recognized as the enzyme that stabilizes fibrin clots by covalently crosslinking specific glutamyl- and lysyl- side chains on assembled fibrin monomers [1, 2]. However, growing evidence suggests FXIII has important functions in mulitiple physiological and pathological processes in addition to hemostasis. In particular, recent evidence suggests that FXIII plays an important role in wound healing and tissue regeneration [3]. Prolonged wound healing has been reported in humans with severe FXIII deficiency [4–7]. Consisted with these reports, animal studies showed that FXIII-deficient mice exhibit delayed cutaneous wound closure relative to FXIII-sufficient mice [8]. A role for FXIII in wound healing is also suggested by studies showing that FXIII-deficient mice exhibit a delay in hepatocyte regeneration following carbon tetrachloride induced liver injury [9]. An impairment in reparative processes has been proposed to explain the cardiac fibrosis observed in aged FXIII-deficient mice [10]. A role for FXIII in the repair of damaged myocardium is also suggested by studies showing an increased incidence of cardiac rupture in FXIII-deficient mice relative to control animals following experimental mycoardial infarction [11].

FXIII activity has also been implicated in the regulation of several inflammatory processes. For example, exogenous FXIII has been shown to limit organ dysfunction resulting from either hemorrhagic shock or gut ischemia/reperfusion injury in rats [12, 13]. In addition, FXIII has been shown to play a role in bacterial clearance in some contexts [14]. Fibrin, the most well-recognized substrate of FXIII, has been shown to promote inflammatory processes in multiple experimental settings, including bacterial infection, experimental encephalomyelitis, arthritis, and colitis [15–20]. Therefore, FXIII could influence inflammation by stabilizing local fibrin matrices. FXIII also crosslinks a variety of other proteins in addition to fibrin, including plasma proteins (i.e., α2-antiplasmin), extracellular matrix proteins (e.g., fibronectin, vitronectin, collagen), and cell surface receptors (e.g., α_V_β_3_ and the vascular endothelial growth factor receptor) [21–24]. Furthermore, intracellular FXIII has been proposed to regulate monocyte/macrophage functions independently of thrombin [25].

Given the potential for FXIII to both promote inflammatory functions and support wound healing/tissue regeneration, it would seem likely that this transglutaminase would play a particularly important role in pathologies characterized by inflammation-mediated tissue damage. A classic example of this is inflammatory bowel disease (IBD), a group of potentially life-threatening disorders characterized by immune dysregulation, inflammatory-mediated damage of gastrointestinal epithelia, and loss of intestinal and endothelial barrier function. FXIII has been indirectly linked to the progression of IBD. Clinical studies have suggested that reduced plasma FXIII levels correlate with active disease in patients with IBD [26–29], but the clincal utility of FXIII as a biomarker in IBD remain to be definitively determined. Moreover, the key question of whether plasma FXIII is simply consumed and depleted in IBD, making it merely a marker of disease, or has a direct role in IBD pathophysiology remains unresolved. The notion that FXIII actively contributes to colitis pathogenesis has gained some support from pilot clinical studies suggesting that FXIII concentrates may be beneficial in IBD [30–33], as well as basic science studies of exogenous FXIII treatment in rats challenged with trinitrobenzenesulfonic (TNBS) acid [34]. In the studies presented here, the hypothesis that FXIII is a determinant of experimental colitis was directly tested using gene-targeted FXIII-A subunit deficient (FXIII^−/−^) mice.

## Materials and Methods

### Animals and statistical analyses

Male 8–12 week old C57BL/6-inbred gene-targeted FXIII-A deficient mice (FXIII^−/−^) [10] and wildtype (WT) littermate controls were used in all experiments. All mice used in these studies were raised in house. The mouse studies presented here were conducted at the Cincinnati Children’s Hospital Research Foundation in accordance with AAALAC guidelines and were approved by the “Cincinnati Children’s Hospital Institutional Animal Care and Use Committee (IACUC).” All mice enrolled in DSS colitis studies were monitored twice daily. Mice were briefly anesthetized with isoflurane (2%) during retro-orbital blood collection and intravenous injections (see below). No other analgesics/anesthetics were used. At harvest, mice were euthanized in accordance with “Cincinnati Children’s Hospital Institutional Animal Care and Use Committee” guidelines by CO_2_ narcosis. Unless otherwise indicated, the data shown represent the mean ± SEM. All *P* values were generated using Prism Graphpad Software as indicated in the text. For analyses of Disease Activity Index data a repeated measures ANOVA was used to account for the intra-correlation within a given mouse. Also included in the analyses were genotype/treatment, time and their interaction. Specific statistical analyses for other data sets are detailed in the text.

### Induction of colitis and treatment with rFXIII

Experimental colitis was induced with 1.5% dextran sulfate sodium (DSS, MW 36000–50000, ICN Biomedicals) in the drinking water as previously described [19]. Unless otherwise specified, the experimental protocol consisted of 7 days of DSS exposure followed by 7 days on normal water. Mice were monitored throughout each experiment for overall appearance and body weight. Freshly collected stool samples were monitored for diarrhea as well as occult blood (Hemoccult Sensa Kit, Beckman Coulter), and overt blood. The sum of a semi-quantitative intestinal bleeding score, stool consistency score and weight loss score was used to generate a Disease Activity Index (DAI) as follows: bleeding score (0—no bleeding, 1—occult blood only, 2—minimal visible blood in feces, 3—evident blood in stool/rectal bleeding); stool consistency score (0—normal, 1—semi-formed, 2—frank diarrhea); weight loss score (0—no weight change, 1—loss of 1 to 5% of body weight, 2—loss of 5 to 10%, 3—loss of 10–15%, 4—loss of >15% of body weight). Recombinant human FXIII-A (rFXIII) produced by Novo Nordisk has been previously described [35, 36]. Mice received either rFXIII (4 mg/kg) or an equal volume of vehicle via an initial intravenous injection the day before DSS challenge followed by an intraperitoneal injection (4 mg/kg) once daily for 14 days starting at the initiation of DSS.

### Histological analyses, cytokines measurements and quantitation of intestinal bacterial translocation

Colonic tissue was fixed in Carnoy’s solution, paraffin embedded and processed for histology and fibrinogen immunostaining as previously described [19, 37]. Colitis severity was assessed using an established semi-quantitative multiparameter histopathological scoring system based on the following criteria: percent area involved (0–4), edema (0–3), ulceration (0–4), crypt loss (0–4), and leukocyte infiltration (0–3) [38]. Colonic cytokine concentrations were determined using Luminex technology on a BioPlex analyzer (Bio-Rad) from homogenates of flash-frozen distal colonic tissue as previously described [39]. Bacterial translocation to the liver was quantitated from homogenates of sterilely harvested liver tissue plated under aerobic (BBL Trypticase Soy Agar with 5% sheeps blood plate) or anaerobic conditions (BBL Brucella Agar with 5% sheeps blood, Vitamin K, and Hemin plate placed inside of a BD GasPak Pouch, BD Bioeciences).

### Measurement of FXIII

Murine plasma samples for FXIII activity measurement were collected from the retro-orbital venous sinus. FXIII activity in mouse plasma was measured using a chromogenic assay (Siemens Healthcare Diagnostics, Germany) following the manufacturer’s instructions. Results are expressed as a percentage of FXIII activity relative to a cohort of unchallenged sex and age matched WT mice from the same colony. Note that the FXIII activity assay does not distinguish between human- or murine-derived FXIII activity. Human rFXIII antigen in mouse plasma was measured by an enzyme immunoassay (Technozym EIA, Technoclone, Vienna) specific for human FXIII that detects both the FXIII-A_2_B_2_ complex and free A_2_ dimer, but does not detect murine FXIII-A or-B. The rFXIII was used as reference material for calibration and control samples.

## Results

### Lack of FXIII impairs disease resolution in murine experimental colitis

**Fig 1:**
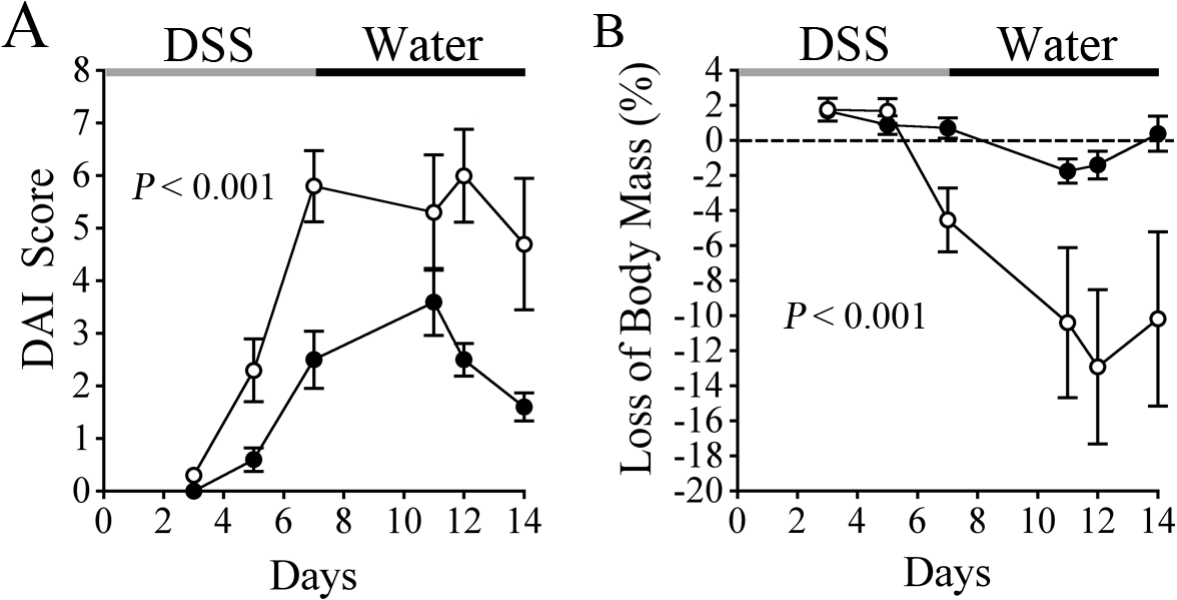
Genetic elimination of FXIII prolongs colitis symptoms. FXIII^−/−^ mice (◯) challenged with DSS develop significantly worse clinical signs of disease relative to WT mice (●) based on a multiparameter Disease Activity Index score (A) and weight loss (B). Note that body mass continued to decline in FXIII^−/−^ mice even after DSS was withdrawn, whereas body mass stabilized and improved in WT animals during this period. *P* values were generated with a 2 way ANOVA, n = 10 mice per cohort.

In order to directly determine if FXIII plays a role in the development and resolution of experimental colitis *in vivo*, FXIII-A deficient (FXIII^−/−^) and WT littermate control mice were enrolled in colitis studies initiated by dextran sulfate sodium (DSS)-induced mucosal damage. The animals were monitored closely throughout the study period using a clinical scoring system based on weight loss, fecal consistency and blood in feces, generating a total disease activity index (DAI, see Methods for details). A worsening in clinical disease characteristics (increase in DAI) was apparent in FXIII^−/−^ mice relative to WT mice by the end of the 7 day DSS challenge period, which was driven primarily by differences in intestinal bleeding (Fig 1A). Dissecting the DAI looking only at intestinal bleeding it was evident that by day 5 of the study, 7 of 10 FXIII^−/−^ mice exhibited intestinal hemorrhage (5 with occult blood in stool and 2 with overtly bloody stools). In contrast, none of the 10 WT mice challenged in parallel exhibited evidence of intestinal bleeding at this early time point (*P* < 0.001, Fisher’s exact test). There was also a trend toward more weight loss in the FXIII^−/−^ cohort within the window of DSS challenge, but this did not reach statistical significance within this 7 day timeframe (Fig 1B). However, FXIII^−/−^ mice showed persistent intestinal hemorrhage as well as continued weight loss *after* withdrawal of the DSS challenge and the return to normal drinking water (Fig 1B). This resulted in a persistently high overall DAI score following the withdrawal of DSS in FXIII^−/−^ mice (Fig 1A). In contrast, WT mice quickly showed a cessation of intestinal bleeding, diarrhea and weight loss when transitioned to normal water, resulting in both a significantly lower and an ultimately declining DAI score following DSS withdrawal compared to FXIII^−/−^ mice. These experiments were completed twice with similar results.

**Fig 2:**
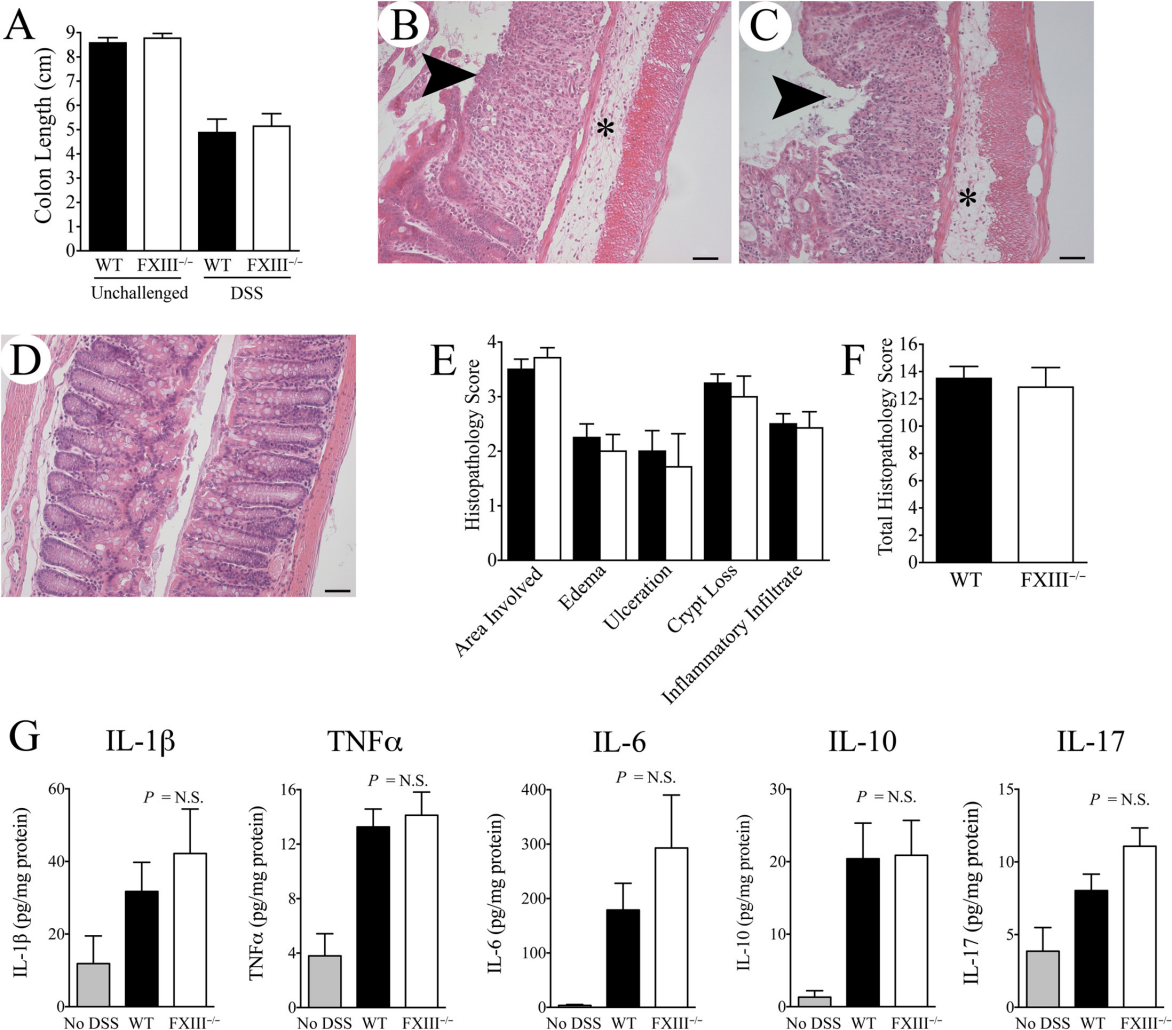
FXIII-deficiency does not alter initial colonic mucosal damage following 7 days of continuous DSS exposure. (A) Colons harvested from FXIII^−/−^ and WT mice immediately after 7 days of DSS challenge (n = 10 per cohort) were significantly shorter than colons harvested from unchallenged mice (n = 4 per cohort), consistent with severe colitis (*P* < 0.01 for each comparison). However, colon lengths were similar in animals of both genotypes following 7 days of DSS exposure indicating comparable levels of initial disease. H&E stained sections of colonic tissue harvested from both WT (B) and FXIII^−/−^ mice (C) had similar evidence of significant mucosal ulceration (arrowheads) and inflammatory edema (*). (D) An unchallenged colon cut in the same plane is shown for comparison. Size bars represent 50 μm. These observations were confirmed using a semi-quantitative multiparameter histopathological scoring system. Shown are the results for each individual parameter (E) and the summation of each parameter yielding a total disease score (F). Black bars and white bars represent WT and FXIII^−/−^ cohorts (n = 10 per cohort), respectively. *P* = N.S for each comparison, Mann-Whitney U test. (G) Shown are the levels of inflammatory cytokines measured in colonic homogenates harvested from WT (n = 12) and FXIII^−/−^ mice (n = 11) immediately following 7 days of DSS challenge as well as levels in colons harvested from unchallenged mice (n = 5). Note that levels of each cytokine measured were significantly elevated in DSS-challenged colons relative to those from unchallenged mice, but FXIII genotype had no significant effect on local cytokine levels.

The reason for the continued worsening in clinical colitis measures observed in FXIII^−/−^ mice following DSS withdrawal was not readily apparent based on multiple detailed analyses of colonic tissue harvested immediately after 7 days of DSS exposure. Here, colons harvested from FXIII^−/−^ and WT mice (n = 8 per genotype) were similarly foreshortened relative to colons harvested from unchallenged FXIII^−/−^ and WT mice, a gross indicator of similar colitis severity in FXIII^−/−^ and WT cohorts given the well-established inverse correlation of colon length and colitis severity (Fig 2A). Consistent with these gross observations, histological examination of colonic tissue revealed similar evidence of inflammatory infiltration, edema, loss of colonic crypts and ulceration based on a multiparameter histological scoring system (Fig 2B–2F). These results suggest that the continued worsening of clinical parameters observed in FXIII^−/−^ mice was not due to more severe initial DSS-induced injury. These results also suggest that the more severe intestinal bleeding observed in FXIII^-/-^ mice relative to WT mice after 7 days of DSS exposure was due to the established bleeding propensity associated with FXIII deficiency, rather than genotype-dependent differences in mucosal damage. Consistent with these more qualitative histological observations, separate quantitative comparisons of multiple inflammatory cytokines known to influence colitis severity (IL-1β, TNFα, IL-6, IL-10, IL-17) in colonic homogenates from FXIII^−/−^ and WT mice following 7 days of DSS exposure were similar between genotypes (Fig 2G). Analyses of intestinal bacterial translocation following 7 days of DSS exposure were also similar in FXIII^−/−^ mice and WT, indicating that the clinical differences observed in FXIII^−/−^ mice following DSS withdrawal were not due to genotype differences in bacterial translocation following mucosal injury. Here, aerobic and anaerobic bacterial growth from homogenates of sterilely harvested liver tissue from FXIII^−/−^ mice and WT mice were compared immediately following 7 days of DSS challenge. Comparable numbers of aerobic and anaerobic CFUs were present in livers from DSS challenged mice of both genotypes (data not shown). As expected, liver homogenates from unchallenged FXIII^−/−^ and control mice had no bacterial CFUs. These data suggest that the continued worsening in clinical status following DSS withdrawal observed in FXIII^−/−^ mice relative to WT mice was not due to more severe DSS-induced colonic mucosal damage, a more robust local inflammatory response, or differences in bacterial translocation.

**Fig 3:**
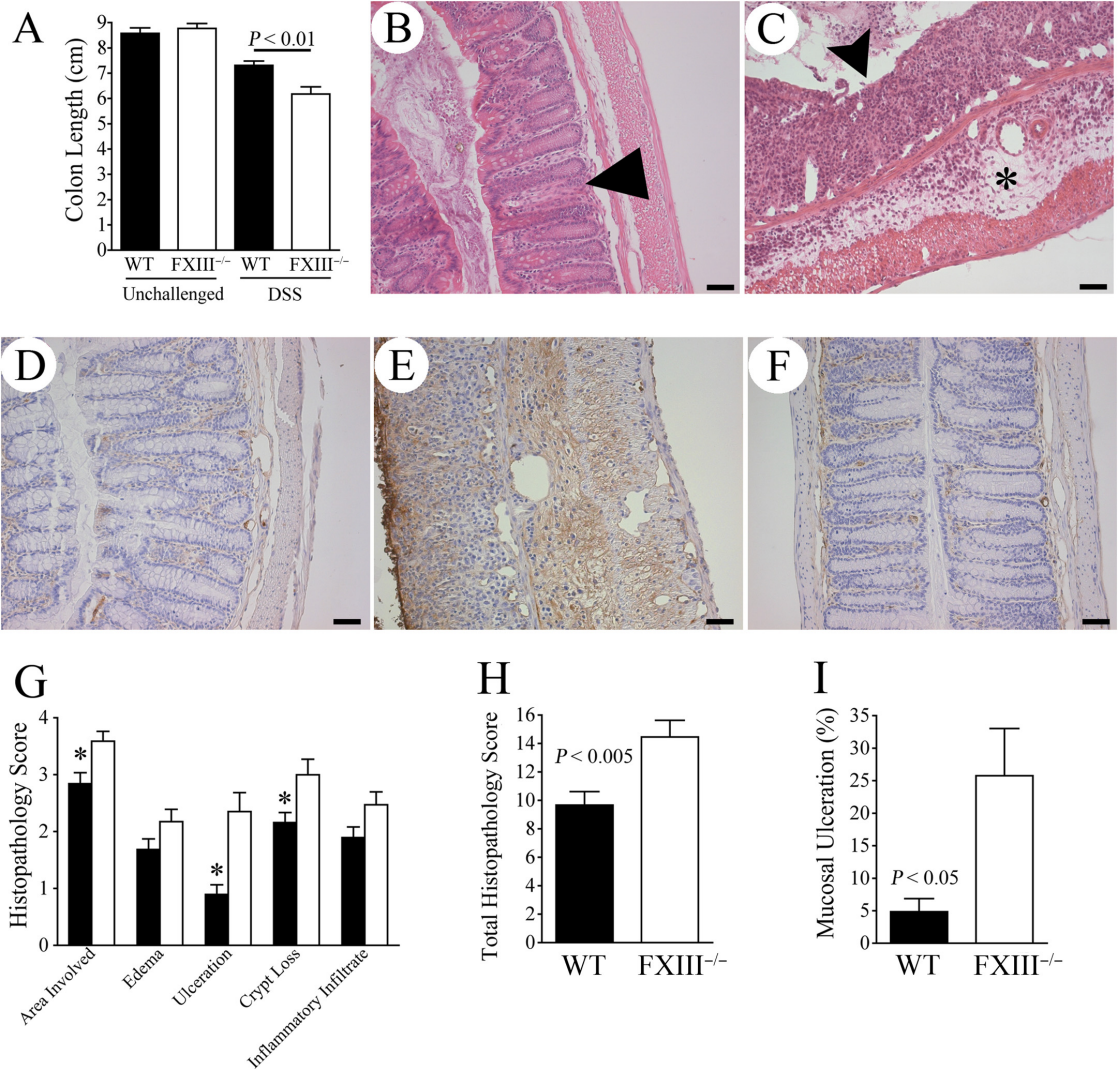
Lack of FXIII results in a failure to resolve colonic damage after DSS exposure. (A) Colon lengths observed in cohorts of WT and FXIII^−/−^ mice 7 days after withdrawal of DSS (n = 10 per cohort). Note that colons were significantly shorter in FXIII^−/−^ mice (consistent with more severe lingering disease) in comparison to WT mice. (B) Representative H&E-stained section of colon tissue harvested from a WT mouse 7 days after DSS withdrawal demonstrated substantial resolution of DSS-induced colitis in this time frame. Note the generally intact mucosa with mild inflammation, and occasional crypt loss and crypt spacing (triangle). (C) In contrast, colons harvested from FXIII^−/−^ mice typically had large areas of ulceration (arrowheads) and inflammatory submucosal edema (*). (D-E) Representative immunohistochemical analyses of fibrin(ogen) deposition (detected by brown staining) within colon tissue sections harvest from WT (D) and FXIII^−/−^ mice (E) 7 days after withdrawal of the DSS challenge. Note that interstitial fibrin(ogen) was minimal in the well-resolved colonic mucosa of WT mice; indeed, the trace fibrin(ogen) detected was generally not appreciably different from that observed in unchallenged WT mice (F). In contrast, interstitial fibrin(ogen) deposits were readily observed in the colons of FXIII^−/−^ mice a full week after withdrawal of DSS, and were particularly intense in the numerous areas of lingering severe mucosal damage. Size bars represent 50 μm. (G-I) Colons harvested from FXIII^−/−^ mice (white bars) had more severe microscopic features of disease relative to WT mice (black bars) in the resolution phase 7 days after withdrawal of DSS challenge based on comparative analyses of histopathology scores evaluating key individual (G) and combined (H) disease scores (n = 10 per cohort). (I) Shown are results of direct microscopic measurement of mucosal ulceration as a percentage of colon length (n = 10 per cohort). * *P* < 0.01, all *P* values were generated with a Mann-Whitney U test.

In contrast to the similarities in overall disease severity observed immediately following 7 days of DSS exposure, significant FXIII-dependent differences in colonic tissue damage were observed in mice 7 days after the withdrawal of the DSS challenge, effectively in the colitis injury “resolution phase.” Here, colons harvested from FXIII^−/−^ mice 7 days after DSS withdrawal were significantly shorter than those harvested from DSS-challenged WT mice (Fig 3A), suggesting more severe colitis in FXIII^−/−^ cohorts. Histological analyses demonstrated significant areas of inflammation, crypt loss and ulceration in colons harvested from FXIII^−/−^ mice 7 days after DSS withdrawal that were similar to those observed immediately after the 7 day DSS challenge. In contrast, the microscopic evidence of colitis apparent immediately following a 7 day DSS challenge had largely resolved in colons harvested from WT animals 7 days following DSS withdrawal. (Fig 3B & 3C). These results suggest that the persistent intestinal hemorrhage and continued weight loss observed in FXIII^-/-^ mice *after* withdrawal of the DSS challenge and the return to normal drinking water was not simply the result of the bleeding tendency associated with FXIII deficiency, but also due to a failure to resolve DSS-induced mucosal damage.

Fibrin(ogen) immunostaining of colonic tissues harvested from FXIII^−/−^ and WT mice 7 days after withdrawal of DSS showed residual fibrin(ogen) deposition within areas of mucosal damage/ulceration, regardless of genotype. Consistent with the observation that FXIII^−/−^ mice had more severe evidence of ongoing or unresolved mucosal pathology than control mice 7 days after DSS withdrawal, qualitatively more fibrin(ogen) deposition was observed in colonic tissue sections from FXIII^−/−^ mice than WT animals challenged in parallel (representative views in Fig 3D and 3E). Predictably, colons from unchallenged mice had essentially no fibrin(ogen) deposition, regardless of genotype (Fig 3F). The FXIII-dependent differences in ulceration, edema, crypt loss and other colonic disease metrics translated into significantly higher histopathological disease scores in FXIII^−/−^ animals relative to WT mice (Fig 3G and 3H). Differences in mucosal ulceration were particularly striking between genotypes. Quantitative microscopic analyses revealed that less than 5% of the colonic tissue harvested from WT mice 7 days after DSS withdrawal exhibited evidence of ulceration, whereas greater than 25% of the colonic mucosa harvested from FXIII^−/−^ mice remained ulcerated (Fig 3I). These results demonstrate that FXIII is an important determinant of the outcome of colitis and becomes particularly important in the period beyond elimination of the underlying insult.

### Plasma levels of FXIII are a highly sensitive marker of inflammatory colitis in mice

**Fig 4:**
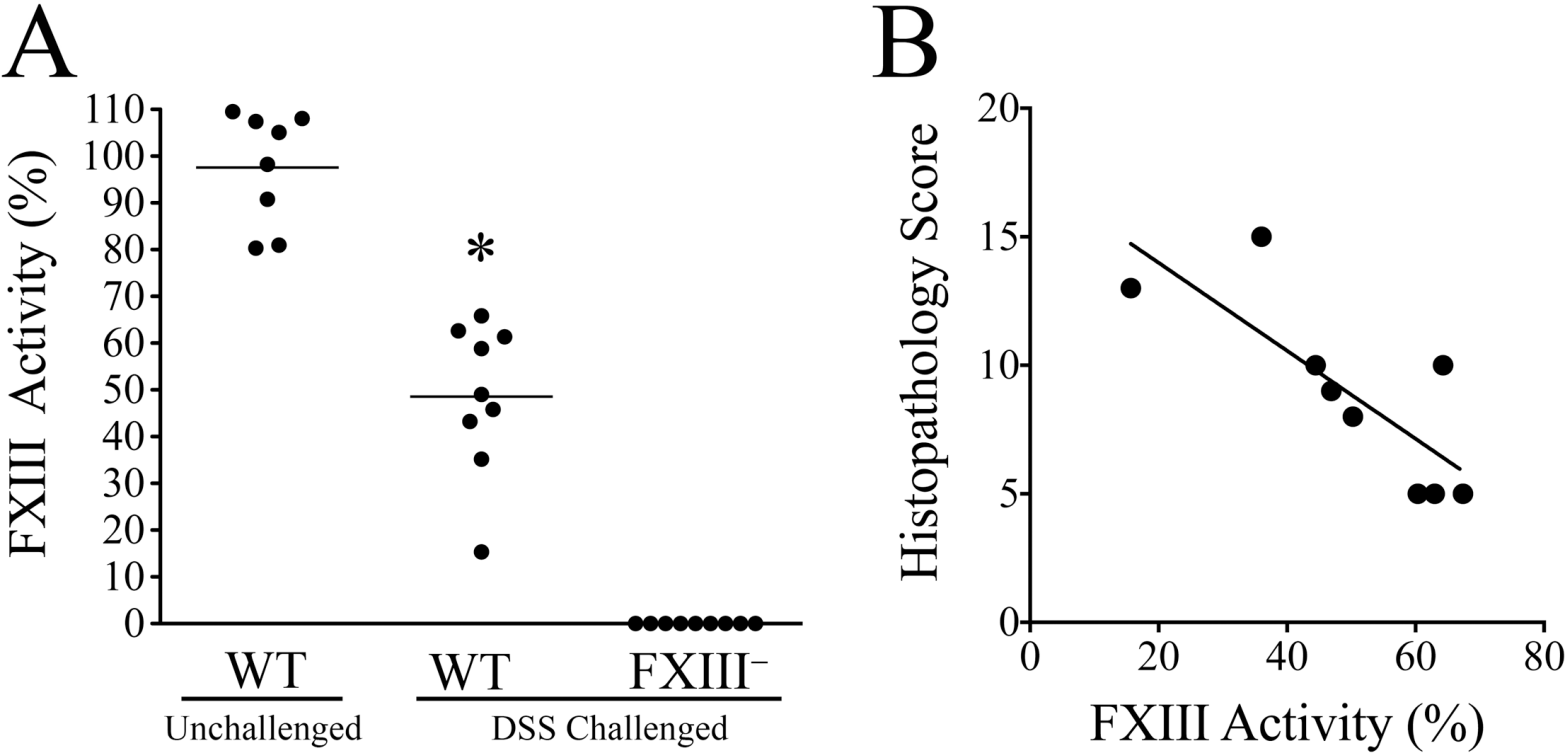
Plasma levels of FXIII are a sensitive biomarker of colitis in mice. (A) Plasma FXIII activity in unchallenged WT mice and DSS-challenged WT and FXIII^−/−^ mice. Plasma samples were collected for analysis 7 days following withdrawal from an initial 7 day DSS-challenge. Note that FXIII activity was significantly lower in DSS-challenged WT mice than unchallenged controls, even well into the colitis resolution phase (* *P* < 0.0001, Mann Whitney U test). Predictably, no FXIII activity was detectable in plasma prepared from FXIII^−/−^ mice. Data were normalized to the unchallenged WT cohort. (B) Plasma FXIII activity in WT mice 7 days after withdrawal of DSS inversely correlated with residual colitis activity as assessed by the histopathology score (R^2^ = 0.63, P < 0.05).

Previous studies have suggested that decreased plasma levels of FXIII may be a useful biomarker in patients with inflammatory bowel disease [26–29]. In order to determine if plasma FXIII activity in mice recovering from experimental colitis correlates with the presence of disease, FXIII activity in plasma from WT and FXIII^−/−^ mice (included as a control) 7 days after DSS withdrawal was compared with values from unchallenged mice of both genotypes. As expected, there was no measurable FXIII activity in plasma harvested from either DSS-challenged or unchallenged FXIII^−/−^ mice (Fig 4A and data not shown). Plasma FXIII activity was significantly decreased in DSS-challenged WT mice relative to unchallenged WT animals (Fig 4A). Moreover, FXIII levels in the DSS-challenged WT mice inversely correlated with disease severity as assessed by histological parameters (Fig 4B). Lower plasma FXIII levels appeared to be particularly predictive of the presence of mucosal ulceration. Only 1 of 4 DSS challenged WT mice with plasma FXIII activity levels > 60% had any histological evidence of ulceration, whereas all of the mice with plasma FXIII activity levels of <60% (5 of 5) manifested some degrees of mucosal ulceration (*P* < 0.05, Fisher’s exact test, two tailed). The inverse correlation of FXIII levels with residual mucosal damage in DSS-challenged mice suggests that increasing FXIII activity could improve mucosal healing in experimental colitis.

### Treatment with rFXIII ameliorates DSS-induced colitis in both FXIII^−/−^ and WT mice

**Fig 5:**
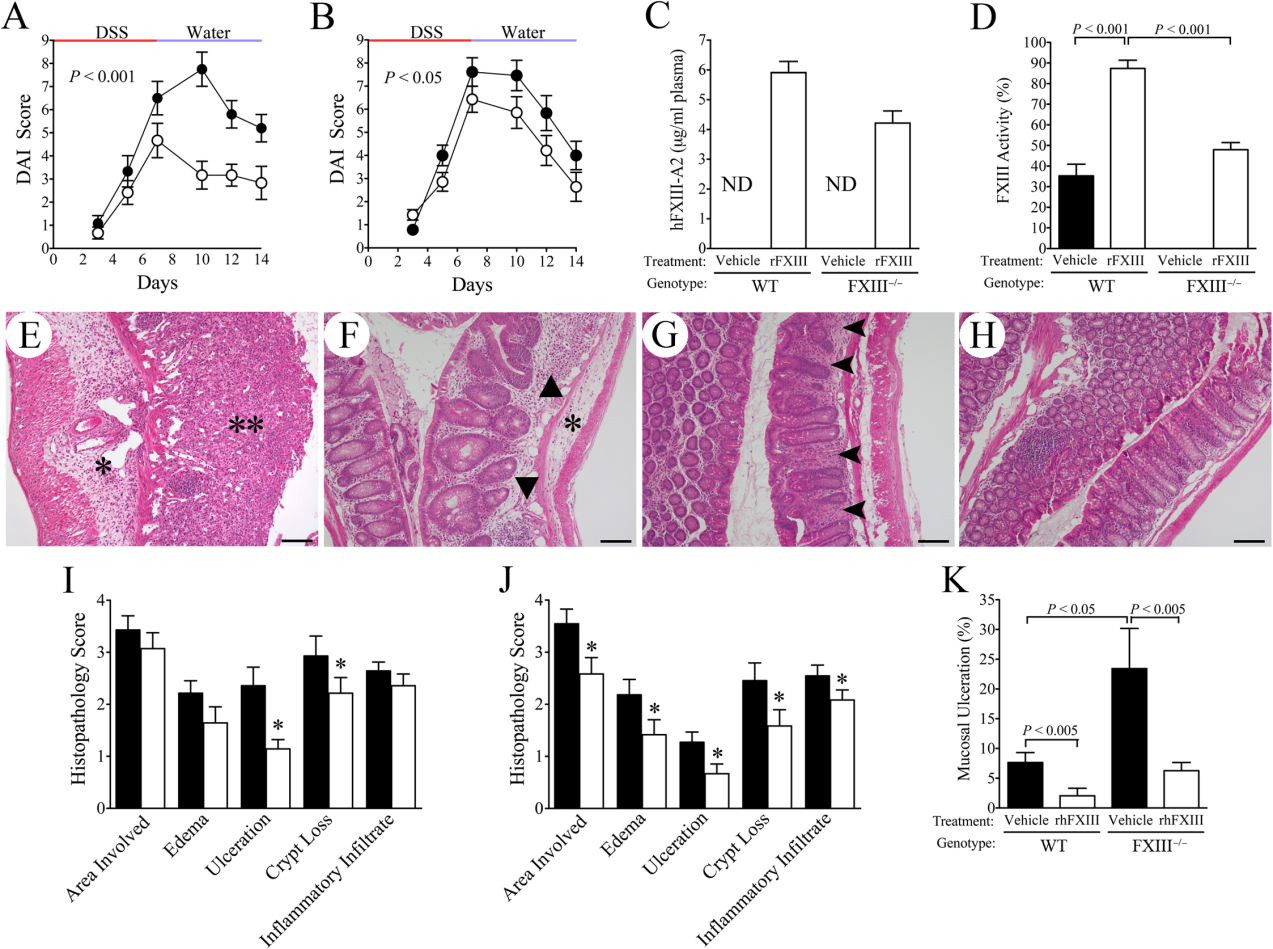
Treatment with rFXIII ameliorates colitis severity in both WT and FXIII^−/−^ mice. (A) Pharmacological treatment of DSS-challenged WT mice with exogenous rFXIII (◯, n = 12) significantly reduced clinical disease parameters (disease activity index, DAI) relative to vehicle treated WT mice (●, n = 11). (B) A more modest, but significant therapeutic benefit of exogenous rFXIII (◯, n = 14) was also observed in FXIII^−/−^ mice relative to vehicle treatment (●, n = 14). (C) Plasma levels of rFXIII measured at the end of the 14 day experiment were comparable between FXIII^−/−^ and WT mice. As expected, no human FXIII protein was detectable (ND; not detected) in vehicle treated mice. (D) Total plasma FXIII catalytic activity measured at the end of the 14 day study period was predictably greatest in rFXIII treated WT mice. Note that this represents activity derived from both endogenous FXIII and exogenous rFXIII). (E) Representative microscopic view of H&E-stained section of colonic tissue harvested at the end of the 14 day study from a vehicle-treated FXIII^−/−^ mouse. Note the large area of inflammatory edema (*) and mucosal ulceration (**). Treatment of FXIII^−/−^ mice with rFXIII significantly limited colonic histopathology (F), but areas of mucosal damage, including ulceration (not shown), crypt loss (triangles) and edema (*) were still common. Consistent with prior results, colons harvested from vehicle-treated WT mice (G) exhibited significantly less evidence of residual pathology relative to vehicle-treated FXIII^−/−^ mice (E) when collected 7 days after withdrawal of the DSS challenge. Nevertheless, small areas of ulceration (not shown) and more frequent areas of crypt spacing associated with inflammatory infiltrates (arrowhead) were still evident. More significantly, DSS-challenged WT mice treated with rFXIII (H) exhibited generally intact, near-normal appearing mucosa. (I) A detailed microscopic evaluation of tissue sections from all FXIII^−/−^ mice employing the multiparameter histopathological scoring system showed that exogenous rFXIII treatment (white bars) resulted in a significant decrease in ulceration and crypt loss relative to vehicle treatment (black bars). (J) Parallel analyses in WT mice revealed a statistically significant diminution in every disease parameter analysed in rFXIII treated WT mice (white bars) relative to vehicle-treated WT mice (black bars). (K) Both FXIII genotype and treatment with rFXIII were important determinants of mucosal ulceration, shown as a percentage of colon length evaluated. Note that cohorts with the least amount of mucosal ulceration also had the highest level of plasma FXIII activity (Compare panels D and K). Bars in E-H represent 100 μm. *P < 0.05, *P* values were generated using a 2 way Anova (A & B) or a Mann-Whitney U test.

In order to determine if rFXIII treatment could ameliorate experimental colitis pathology, cohorts of WT and FXIII^−/−^ mice were challenged with 7 days of DSS followed by water for 7 days and treated throughout the study period once daily with either 4 mg/kg rFXIII or an equivalent volume of vehicle (n = 11 to 15 per cohort). This dosing regimen was based on pharmacokinetic data that determined the half-life of rFXIII to be approximately 30 hours whether rFXIII was administered through intravenous or intraperitoneal injections. Furthermore, the half-life of rFXIII was the same in healthy and DSS-challenged mice (see S1 File for details). Consistent with previous reports showing that stress, including daily investigator handling of animals, exacerbates DSS-induced colitis severity [40, 41], DAI scores were appreciably higher in mice of both genotypes undergoing a daily injection regime over mice merely placed on DSS. Despite the more aggravated course of disease associated with the experimental design, exogenous rFXIII supplementation significantly ameliorated overall colitis disease relative to vehicle-treated animals based on both overall clinical DAI scores and multiparameter histological disease metrics (Fig 5). Note that the data shown in Fig 5 represent the compiled data from three experiments, each with similar results. The most impressive improvement in colitis outcome in mice treated with exogenous rFXIII was actually observed in WT cohorts that experienced a partial loss of FXIII following DSS challenge but still retained the capacity to generate endogenous FXIII (Fig 5A). A statistically significant improvement in disease outcome was also observed in rFXIII-treated FXIII^−/−^ cohorts (Fig 5B). However, with the rFXIII dosing regimen employed the phenotypic benefit of exogenous rFXIII was more modest in FXIII^−/−^ mice than that appreciated in WT cohorts, possibly due to an inability to more effectively restore FXIII levels in mice devoid of any endogenous FXIII.

Measurements of total FXIII activity and rFXIII antigen in plasma obtained at the end of the study period corresponded well to predictions based on the endogenous FXIII activity in WT mice with colitis and to previous pharmacokinetics data (Fig 5C & 5D). Specifically, DSS-challenged FXIII^−/−^ mice treated with rFXIII exhibited total plasma FXIII activity levels that were approximately 50% of unchallenged control mice, whereas DSS-challenged WT mice treated with rFXIII had activity levels comparable to unchallenged mice (Fig 5D). Note that the FXIII activity assay used in these studies does not distinguish between endogenous murine FXIII and human-derived rFXIII. Therefore, FXIII activity in WT mice represents the combination of native murine FXIII and administered rFXIII, whereas in FXIII^-/-^ mice with no endogenous FXIII, any FXIII activity represents only administered rFXIII (Fig 5D). In contrast, the assay used to measure FXIII antigen is specific for human FXIII, and therefore is representative only of administered rFXIII in either genotype (Fig 5C). Histological analyses of colonic tissue harvested at the completion of the study showed that treatment with rFXIII resulted in a significant improvement in the histopathological findings of colitis relative to vehicle treatment in both FXIII^−/−^ and WT mice (see representative colonic tissue sections in Fig 5E–5H). Qualitatively, the most severe histopathological evidence of disease, including large areas of mucosal ulceration and inflammatory edema, were observed in vehicle treated DSS-challenged FXIII^−/−^ mice (Fig 5E). Treatment with rFXIII-A significantly limited the degree of crypt loss and mucosal ulceration relative to vehicle treatment in FXIII^−/−^ mice, but areas of mucosal ulceration, crypt loss, and inflammatory edema were still readily discernible (see Fig 5F for representative section and Fig 5I for semi-quantitative evaluation of individual disease metrics). While not as severe as the histopathology observed in vehicle treated FXIII^−/−^ mice, vehicle treated WT mice also had numerous areas of inflammatory infiltration, crypt loss, and edema and a modest degree of ulceration (see Fig 5G for representative section and Fig 5J for semi-quantitative evaluation of individual disease metrics). It is notable that WT mice treated with rFXIII exhibited a significant improvement in virtually every disease parameter evaluated in the multiparameter histopathological scoring system relative to vehicle treated WT mice (Fig 5H & 5J). Overall, the most striking genotype/treatment related differences were seen in measurements of complete mucosal ulceration as a function of colon length, where the cohorts with the highest plasma FXIII activity were noted to have the least degree of mucosal ulceration (compare Fig 5D and 5K). In fact, ~40% (5 of 12) of the rFXIII treated WT mice and ~21% (3 of 14) of the rFXIII treated FXIII^−/−^ mice had no evidence of ulceration whatsoever, whereas microscopic evidence of ulceration was present in 100% of vehicle treated mice of both genotypes (*P* < 0.05 for each comparison, Fisher’s exact test, two-tailed).

## Discussion

These studies indicate that FXIII is an important determinant of experimental colitis pathophysiology. The genetic elimination of FXIII did not appear to significantly alter the initial development of DSS-induced mucosal ulceration, weight loss and other microscopic and clinical disease metrics. However, the presence of FXIII greatly improved disease outcome following the withdrawal of the DSS challenge in WT mice. The genetic elimination of FXIII transglutaminase function resulted in ineffective resolution of colitis in mice transiently challenged with DSS, resulting in persistent colonic mucosal damage following DSS challenge. These data suggest that FXIII strongly supports reparative processes following colonic damage and secondary inflammatory injury. These studies also showed that active colitis is strongly associated with significantly reduced plasma levels of FXIII in mice, presumably due to consumption resulting from local thrombin activation of FXIII or local margination associated with fibrin matrix deposition. This finding parallels previous studies in human subjects with IBD that suggested an inverse correlation between circulating FXIII levels and colitis severity [26–29, 42]. However, more recent clinical study data (http://clinicaltrials.gov/ct2/show/results/NCT01706159) could not confirm a correlation between FXIII activity and disease activity in patients with ulcerative colitis. Nevertheless, the animal studies presented here showed that pharmacological strategies designed to partially restore FXIII significantly ameliorated experimental colitis pathology in both FXIII^−/−^ and WT mice. These results demonstrate that the loss of FXIII in colitis-challenged WT mice is not merely a marker of disease, but actively contributes to disease pathogenesis.

The observed failure to efficiently resolve colonic mucosal ulceration and reactive inflammatory processes in the absence of FXIII could be explained, at least in part, by a role for FXIII in mucosal wound repair in the context of colitis. The view that FXIII could play an important role in the repair of mucosal damage in colitis is consistent with studies showing that FXIII is present in the matrix of damaged colonic tissue in humans with ulcerative colitis [42]. This conclusion is also consistent with previous reports linking FXIII to wound repair in other experimental contexts. The general importance of FXIII to tissue repair was directly documented through studies showing that FXIII^−/−^ mice exhibited delayed repair of excisional skin wounds [8], and a delay in hepatic repair following carbon tetrachloride-induced intoxication of centrilobular hepatocytes [9]. Furthermore, altered reparative processes within cardiac tissue has been proposed to underlie the hemosiderin deposition and fibrosis observed within the hearts of aged FXIII^−/−^ mice as well as the increased incidence of fatal left ventricular rupture in FXIII^−/−^ mice following myocardial infarction [10, 11].

FXIII could contribute to both reparative and inflammatory processes in the colon through multiple nonmutually exclusive mechanisms. One well-established function of this transglutaminase is the covalent crosslinking of fibrin monomers in newly assembled polymers. Loss of FXIII-mediated fibrin crosslinking results in both less stable provisional fibrin matrices and impaired platelet-mediated clot retraction [1, 43]. Given that mucosal ulceration and hemorrhage are hallmarks of inflammatory colitis, one attractive mechanism by which FXIII could limit colitis severity is by supporting fibrin-mediated hemostatic function and fibrin-supported tissue reorganization events. However, other distinct fibrin-dependent mechanisms may link FXIII activity to colitis outcome. It has previously been shown that leukocyte engagement of fibrin(ogen) through the leukocyte integrin receptor α_M_β_2_ drives colitis severity, apparently by supporting counterproductive local inflammatory cell activation events [19]. Specifically, it was established that gene-targeted mice carrying a mutant form of fibrinogen that retained clotting function but lacked the α_M_β_2_ binding motif (Fibγ^390-396A^) were highly protected from DSS-induced mucosal damage relative to mice carrying wildtype fibrinogen [19]. More recent studies have suggested that the alteration in the α_M_β_2_ binding motif of the fibrinogen γ chain in Fibγ^390-396A^ also limits FXIII binding to fibrinogen and delays FXIII-mediated fibrin crosslinking [44]. The data presented here would suggest that the previously observed protection from DSS-induced mucosal damage conferred by the Fibγ^390-396A^ mutation [19] is not due to loss of FXIII function in these animals. Indeed, it is possible that a dominant function of FXIII in altering colitis outcome may be indirectly related to limiting fibrin-based inflammatory mechanisms. Under this model, loss of FXIII in colitis would result in a continuous cycle of local fibrin deposition and rapid dissolution, ineffective resolution of tissue damage due to fibrin matrix instability, and, counter-intuitively, prolonged fibrin deposition within the colonic mucosa driving pathological inflammatory events. This notion is consistent with the observation presented here that colons harvested from FXIII^−/−^ mice exhibit evidence of persistent fibrin deposits relative to wildtype mice well after the discontinuation of the DSS challenge. Defining the precise relationship between FXIII and fibrin in the resolution of colitis is experimentally approachable using mice with selected functional deficits in both fibrinogen and FXIII, and studies of this type constitute an attractive future direction. Of course, any alteration in FXIII-mediated fibrin clot stabilization at sites of mucosal ulceration may simultaneously alter vascular integrity, colonic barrier function, reparative processes and inflammatory events. Regardless of the precise individual benefits and liability of FXIII function in colitis, the present studies directly show that retaining or boosting FXIII levels, in balance, improves the overall outcome of colitis in experimental animals.

The present studies establishing the importance of FXIII in colitis are consistent with earlier findings pointing to a role for other hemostatic factors in the progression of this often devastating disease [19, 45, 46]. However, the current studies do not formally exclude the possibility that FXIII influences colitis through mechanisms independent of hemostasis *per se*. Indeed, FXIII can crosslink other matrix and plasma proteins beyond fibrin monomers, including vitronectin, fibronectin, collagen, von Willebrand factor and α_2_-antiplasmin [21, 22], and many of these proteins are easily coupled to reparative processes *in vivo*. FXIII also has been proposed to directly regulate macrophage and platelet function through intracellular mechanisms coupled to cytoskeletal elements and cell-associated signaling molecules (e.g., angiotensin type I receptor (AT_1_R) [47, 48]. The possibility that FXIII could influence intracellular events important in macrophage function was also suggested by studies showing that macrophages from FXIII-deficient patients exhibit diminished receptor-meditated phagocytosis *in vitro* relative to macrophages prepared from normal control subjects [49]. However, any proposed fibrinogen-independent intracellular or extracellular mechanisms linking FXIII to colitis and other pathological and physiological processes remain to be definitively tested in an *in vivo* setting.

A compelling body of evidence points to colonic microflora, particularly the inopportune translocation of resident intestinal flora following epithelial tissue disruption, as central to the pathogenesis of colitis in both humans and mice [50, 51]. Thus, another mechanism by which FXIII could positively influence the outcome of colitis is by supporting the containment and ultimate clearance of intestinal microorganisms within breached and inflamed colonic tissues. There is strong and direct evidence that FXIII contributes to an effective host antimicrobial response, including studies in FXIII^−/−^ mice indicating that FXIII transglutaminase supports bacterial clearance in the setting of streptococcal skin infection [14]. Here, FXIII function was proposed to support bacterial clearance, at least in part, by crosslinking bacterial surface proteins to fibrin [14]. This hypothesis is consistent with myriad other studies showing that host fibrin(ogen) is a significant factor in both bacterial virulence and host clearance of microbial pathogens in multiple contexts [15, 18, 20, 52–54]. Thus, when intestinal barrier function is compromised, FXIII-mediated fibrin crosslinking reactions could limit colitis severity by supporting microbe immobilization (limiting dissemination of colonic microflora) and, ultimately, the clearance of any translocated bacteria within tissues [50]. However, the findings here that the loss of FXIII had no significant impact on bacterial translocation following DSS challenge would suggest that FXIII-mediated events supporting bacterial clearance are not a defining factor in colitis severity in this context.

In summary, the results of the present studies indicate that FXIII plays an important supportive role in the repair of colitis-induced mucosal damage in mice. The genetic elimination of FXIII resulted in a severe impairment in the resolution of mucosal damage following colitis challenge, whereas dosing of exogenous rFXIII significantly ameliorated experimental colitis pathology.

## Supporting Information

S1 File Pharmacokinetics of rFXIII in WT and FXIII^−/−^ mice. (PDF)
