# Supplementary material for: Factor XIII Transglutaminase Supports the Resolution of Mucosal Damage in Experimental Colitis
Source: PLoS One. 2015 Jun 22;10(6):e0128113. doi: 10.1371/journal.pone.0128113 (PMC4476663; doi:10.1371/journal.pone.0128113)
Supplement: S1 File — (PDF) [file pone.0128113.s001.pdf]

Pharmacokinetic analysis of recombinant human FXIII-A (rFXIII) in wildtype and FXIII$^{-/-}$ mice.

As a prelude to studies evaluating if treatment with exogenous FXIII could improve experimental colitis pathology, we performed pharmacokinetic analyses of a recently described recombinant human FXIII-A subunit (rFXIII) in WT and FXIII$^{-/-}$ mice [35,36]. The goal of these analyses was to define the optimal rFXIII dose and administration schedule that would raise plasma FXIII activity in FXIII$^{-/-}$ mice to levels comparable to those observed in WT mice. Notably, previous studies have shown that a human plasma-derived FXIII is active in mice [11,23]. Furthermore, there is an excess of free FXIII-B subunits in plasma (50% of the available FXIII-B circulates unbound to FXIII-A in humans) and endogenous murine FXIII-B subunit can efficiently complex with rFXIII (data not shown).

The half-life of rFXIII in mice was determined to be approximately 30 hours when complexed to FXIII-B (Supplemental Tables 1 and 2) and 1 hour if free (data not shown), and was not affected after repeated dosing. The half-life was consistent regardless of whether the injections were intravenous or intraperitoneal, and the bioavailability after intraperitoneal injection was close to 100 % , data not shown). Furthermore, the half-life of rFXIII was the same in healthy and DSS-challenged mice (data not shown). As there is no mouse FXIII-B subunit specific antibody, plasma FXIII-B subunit levels in WT and FXIII-A subunit deficient mice were indirectly determined by comparing the PK profile of dosed rFXIII. The PK profile was similar between genotypes, indicating that WT and FXIII$^{-/-}$ mice carry comparable plasma levels of free FXIII-B (Supplemental Tables 1 and 2). Simulations of different dosing regimens showed that daily dosing of FXIII$^{-/-}$ mice with 4 mg/kg i.p. rFXIII would result in plasma trough levels of ~55% (relative to the activity in unchallenged WT mice), the maximum levels achievable based on the availability of free FXIII-B subunits (see Supplemental Figure). Therefore, complete reconstitution of FXIII activity in FXIII$^{-/-}$ mice to levels found in unchallenged WT mice was not possible. Nevertheless, we hypothesized that an increase of FXIII level with ~55% of FXIII activity would be sufficient to significantly ameliorate DSS-induced colitis in FXIII$^{-/-}$ mice as well as improve disease outcome in DSS-challenged WT mice.

S1 Table. Pharmacokinetic parameters of single dose rFXIII in WT and $FXIII^{-/-}$ mice.

Mouse Genotype	Dose (mg/kg)	C_{max} (μg/ml)	AUC (h*μg/ml)	$T_{½}$ (h)	MRT (h)
**Wildtype**	4	12.8	253	34.9	44.7
**$FXIII^{-/-}$**	4	13.0	220	30.9	36.2

Shown are the pharmacokinetic parameters after single dose intravenous administration of rFXIII as estimated by noncompartmental analyses based on mean plasma human FXIII-A antigen concentration versus time. C_{max} – maximal plasma concentration, AUC – Area Under the Curve, T½ – plasma half-life, MRT – mean resident time

S2 Table. Pharmacokinetic parameters of multiple dose rFXIII in WT and $FXIII^{-/-}$ mice.

Mouse Genotype	C_{max} (μg/ml)	AUC (h*μg/ml)	T½ (h)	MRT (h)	Vss (ml/kg)
Wildtype	8.08	277	34.4	47.5	600
$FXIII^{-/-}$	10.0	295	32.0	44.9	532

Shown are the pharmacokinetic parameters after intravenous administration of rFXIII for 5 doses every 48 hours at 4 mg/kg/dose as estimated by noncompartmental analyses based on mean plasma human FXIII-A antigen concentration versus time. C_{max} – maximal plasma concentration, AUC – Area Under the Curve, T½ – plasma half-life, MRT – mean resident time, Vss – Volume of distribution at steady state.

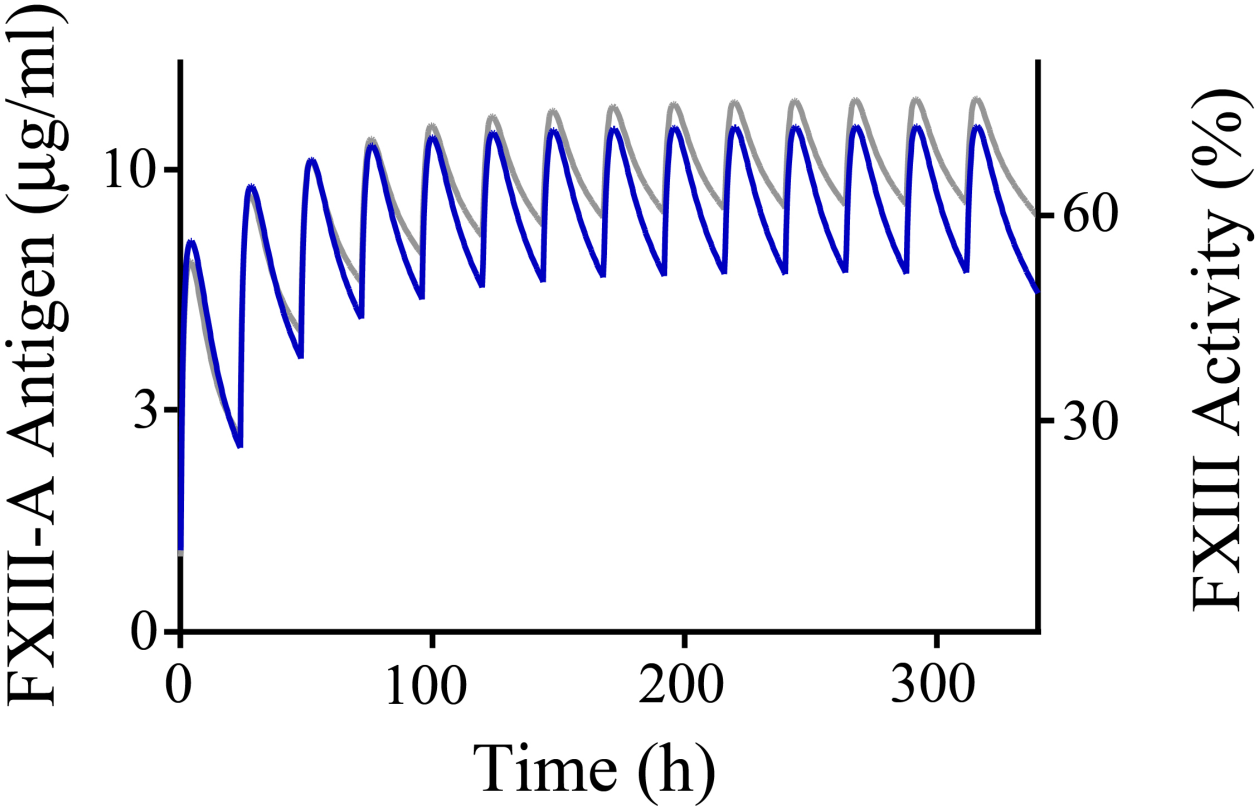

Supplemental Figure. Predicted pharmacokinetic profile of rFXIII in WT and FXIII$^{-/-}$ mice. Shown is the expected PK profile of rFXIII after once daily i.p. dosing of 4 mg/kg in WT (gray) and FXIII$^{-/-}$ (blue) mice. FXIII$^{-/-}$ mice reached a trough level corresponding to ~50% FXIII activity after 3 doses. Note that the predicted FXIII activity does not take into account the endogenous murine FXIII present in WT mice.
